# Association of *MTHFR* C677T and *MTRR* A66G Gene Polymorphisms with Iranian Male Infertility and Its Effect on Seminal Folate and Vitamin B12

**DOI:** 10.22074/IJFS.2021.6155

**Published:** 2021-01-27

**Authors:** Mozhgan Raigani, Niknam Lakpour, Meysam Soleimani, Behrooz Johari, Mohammad Reza Sadeghi

**Affiliations:** 1 Biotechnology Research Center, Pasteur Institute of Iran, Tehran, Iran; 2 Reproductive Biotechnology Research Center, Avicenna Research Institute, ACECR, Tehran, Iran; 3 Department of Pharmaceutical Biotechnology, School of Pharmacy, Hamadan University of Medical Sciences, Hamadan, Iran; 4 Department of Medical Biotechnology and Nanotechnology, School of Medicine, Zanjan University of Medical Science, Zanjan, Iran; 5 Monoclonal Antibody Research Center, Avicenna Research Institute, ACECR, Tehran, Iran

**Keywords:** Folate, Male Infertility, *MTHFR*, *MTRR*, Vitamin B12

## Abstract

**Background:**

The relation between key enzymes in regulation of folate metabolism and male infertility is the subject of numerous studies. We aimed to determine whether 5, 10-methylenetetrahydrofolate reductase (*MTHFR*) C677T and methionine synthase reductase (*MTRR*) A66G genotypes are associated with male infertility in Iranian men and to evaluate its effect on seminal levels of folate and vitamin B12.

**Materials and Methods:**

In this retrospective study, semen and peripheral blood samples were collected from 254 men with oligoasthenoteratozoospermia (OAT) and 77 normozoospermic men who attended Avicenna infertility clin- ic. Single nucleotide polymorphism (SNP) analysis was carried out in genomic DNA by polymerase chain reaction (PCR)-restriction fragment length polymorphism (RFLP) method for *MTHFR* C677T and *MTRR* A66G gene poly- morphisms.

**Results:**

In *MTHFR* C677T, our founding showed that T carrier was conversely lower in OAT than normozoospermic men (χ^2^-test=7.245, P=0.02) whereas in *MTRR* A66G, A and G carrier showed no significant difference between the two groups (χ^2^-test=1.079, P=0.53). The concentration of seminal folate was not different between normozoospermic (18.83 ± 17.1 ng/ ml) and OAT (16.96 ± 14.2 ng/ml) men (P=0.47). The concentration of vitamin B_12_ was slightly higher in normozospermic men (522.6 ± 388.1 pg/ml) compared to OAT men (412.9 ± 303.6 pg/ml, P=0.058).

**Conclusion:**

The MTHFR C677T and MTRR A66G have no effect on the concentrations of seminal folate and vitamin B_12_. The present study showed that two SNPs of *MTRR* A66G and *MTHFR* C677T cannot be seen as a risk factor for male factor subfertility.

## Introduction

Infertile men are adult individuals who fail to achieve pregnancy after one year of having intercourse without any birth control. Almost 50% of fertility problems are related to male factors and most of the affected men exhibit low sperm quality (1). Folates participate in DNA, RNA and histone methylation reactions -via involvement in homocysteine metabolism-which can be involved in spermatogenesis (2). 5, 10-methylenetetrahydrofolate reductase (MTHFR) is the basic regulatory enzyme of folate metabolism.* MTHFR* gene is located on chromosome 1 (1p36.22) (3). Methionine synthase reductase (*MTRR*) gene, on chromosome 5, also plays a vital role in DNA synthesis. It is well known that folate and methionine metabolism play essential roles in both DNA methylation and synthesis (4).

MTHFR and MTRR play key interrelated roles in folate metabolism. MTHFR catalyzes the regulation of cellular methylation through the conversion of 5, 10-methylene tetrahydrofolate (THF) to 5-methyl-THF, the primary circulating form of folate metabolism. MTRR is required for the reductive methylation of vitamin B12, also known as cobalamin, an activated cofactor for methionine synthase (MTR), which catalyzes the methylation of homocysteine to methionine (5).

There are two important polymorphisms in the* MTHFR* gene; C677T (6) and A1298C (7) mutations which can affect its biological activity. Numerous researchers have focused on a C>T mutation at nucleotide position 677 in exon 3 of the* MTHFR* gene which changes alanine to valine (8). The* MTHFR* C677T mutation decreases the corresponding protein activity, so that it impairs the ability to process folate. This mutated gene leads to increase amino acid homocysteine level (9).* MTHFR* polymorphisms are prevalent in terms of the local area C677T and its distribution differs among different population. The prevalence of heterozygous and homozygous state is about 40% and 10% in Caucasians, respectively (10).

The homozygous C667T in the* MTHFR* gene is reported to be associated with the risk of a number of diseases in humans, including some cardiovascular abnormalities (atherosclerosis, cardiovascular) (11), cancers and neural tube disorders (4). Previous studies have shown that activity of MTHFR is much higher in testis instead of the other major organs in adult male mouse. So it was suggested that might play an important role in spermatogenesis (12).

One of the most prevalent polymorphisms in the *MTRR* gene is A66G, which results in an amino acid substitution from methionine to isoleucine at codon 22 (M22I) (4). *MTRR* 66A>G also affects conversion of homocysteine to methionine, which adversely influences enzyme activity and thus is considered as a genetic risk factor for hyperhomocysteinemia. *MTRR* A66G polymorphism may also induce DNA hypomethylation by regulating homocysteine (Hcy) levels (5).

Functional studies indicated that individuals possessing both mutations showed the lowest enzyme activities (4). As a result, both DNA methylation and DNA synthesis may be altered by interacting with homocysteine, vitamin B12 and folate (13). Polymorphisms in the* MTHFR* gene, 677C>T, 1298A>C and the *MTRR* gene, 66A>G, are associated with male infertility

In the previous study, we demonstrated that administration of folic acid and co-administration of folic acid and zinc sulphate during randomized, double-blind dietary program did not improve the quality of sperm in infertile men with severely compromised sperm parameters, oligoasthenoteratozoospermic (OAT) (1). The present study aimed to assess and compare the mutations in* MTHFR* gene 677C>T, as well as *MTRR* gene 66A>G in men with OAT and normozospermia. Furthermore, we investigated the correlation of these genetic variants with seminal folate and vitamin B12 levels. 

## Materials and Methods

### Patients

In this retrospective study, semen analysis was done for 254 men with OAT and 77 men with normozospermia who attended Avicenna Infertility Clinic (AIC; Tehran, Iran). In all samples, semen parameters, sperm concentration, sperm motility, viability and morphology were evaluated in accordance with the WHO guidelines (14) and they were classified as normozoospermic and OAT samples. Infertile men with sperm concentrations of <20×10^6^ ml^-1^, sperm motility <50% (grades a, b, c) and normal sperm morphology <30% were included as OAT and infertile men with normal sperm parameters were classified as normozospermia.

Patients with leukocytospermia (leukocyte concentration greater than 1×10^6^ /ml), varicocele, chronic systemic diseases, autoimmune disorders or history of smoking, in addition to excessive alcohol and drug consumption were excluded from the study.

### Ethical approval

Each participant provided a written informed consent before the collection of their biological samples. Additionally, the study was approved by Ethics Committee of the Avicenna Research Institute. The ethics code (85.3496) was allocated for our project. All procedures were done in accordance with the ethical Helsinki standards. 

### Preparation of semen samples 

The semen samples were collected by masturbation only 48-72 hours after sexual abstinence and ejaculated into a clean plastic specimen cup. Samples were delivered to the laboratory within 1 hour of collection at 20-40°C. Following complete liquefaction, standard semen analysis was conducted for all participant according to WHO guidelines (14). The remnant semen was centrifuged at 2000 rpm for 5 minutes and supernatant was divided in several aliquots and stored at -20°C for future analysis.

### Blood sampling and DNA extraction

Genomic DNA was extracted from white blood cells according to sodium salting out extraction method. The DNA purity and concentrations were determined by measurement of absorbance at 260/280 nm.

### Genotyping* MTHFR* C677T

The C677T SNP (rs#1801133) of* MTHFR* gene was studied by polymerase chain reaction (PCR)-restriction fragment length polymorphism (RFLP) method. 

We employed the

F: 5'-CATCCCTATTGGCAGGTTACCC-3' and 

R: 5'-TGCGAGGACGGTGCGGTGAGA-3'

PCR primers which were designed using Gene Runner software. PCR cycles were as follow: 32 cycles of 94°C for 30 seconds, 58.4°C for 30 seconds and 72°C for 30 seconds. A 271 bp PCR product was digested overnight by HinfI restriction enzyme endonuclease (New England Biolabs, USA) with the recognition sequence 5'-GˆAGTC-3' at 37°C. The PCR products were visualized on 2% agarose gel. A 271 bp fragment was seen in the absence of mutation [wild-type (CC)], 271, 177 and 94 bp fragments were observed in the presence of one allele mutation [heterozygotes (CT)], and 177 and 94 bp fragments were seen in the presence of two allele mutations [homozygotes (TT)].

### Genotyping *MTRR* A66G

The PCR-RFLP assay was employed for identifying *MTRR* A66G polymorphisms. 

The primers for *MTRR* A66G: 

F: 5'-AGGCAAAGGCCATCGCAGAAGACAT-3' and

R: 5'-GGCTCTAACCTTATCGGATTCACTA-3' 

were designed using Gene Runner software. The forward primer was comprised of a mismatch (underlined base C in the primer sequence), generating an NdeI restriction site, 5'-CAˆTATG-3', when the polymorphic allele was present. PCR cycles were as follow: 35 cycles of 94°C for 30 seconds, 58.4°C for 30 seconds and 72°C for 30 seconds. The anticipated PCR product of 98 bp was digested into fragments of 74 and 24 bp by NdeI restriction enzyme endonuclease (NEB, USA) in the presence of the G allele but remains uncut in the presence of the A allele (15). It means, a 98 bp fragment was seen in the absence of mutation [wildtype (AA)], 98, 74 and 24 bp fragments were observed in the presence of only one mutated allele [heterozygotes (AG)], and 74 and 24 bp fragments were seen in the presence of two mutated alleles [homozygotes (GG)].

### Measurement of seminal vitamin B12 and folate concentration 

Semen folate and vitamin B_12_ levels were measured using radioimmunoassay (RIA) method according to the manufacturer’s instructions (MP BiomedicalsSimulTRAC-SNB Radioassay Kit VITAMIN B12[57Co]/ folate[^125^I]; MP Biomedicals, USA).

### Statistical analysis

Results of the two groups were compared using SPSS 16.0 for Windows software (SPSS Inc, Chicago, IL, USA). TwoSample Kolmogorov-Smirnov test (K-S test) revealed that our data follow a normal distribution and thus parametrical test was employed. Monte Carlo test was conducted to compare* MTHFR* C>T and *MTRR* A>G mutations in normozoospermic and OAT groups. Folate and vitamin B12 concentrations in two studied groups were compared using parametric tests such as Independent-Samples t test and unconditional logistic regression model to calculatethe odds ratios (OR) and 95% confidence intervals (95% CI). Fisher’s exact test was employed in analyzing association of* MTHFR* and *MTRR* mutations with semen concentration of folate and vitamin B12. Differences were considered significant if P value was<0.05. 

## Results

Demographic characteristic information, including age and infertility period in OAT and normozoospermia men, are given in Table 1, showing that there is no significant difference between them. Therefore, they could not be considered as confounding effect. 

**Table 1:** Demographic characteristic information


Groups	Number	Age (Y)	Length of infertility (Y)

Oligoasthenoteratozoospermic group	254	37.07 ± 7.26	5.89 ± 6.4
Normozoospermic group	77	32.33 ± 4.03	5.24 ± 5.5
t test, P value		-1.59, 0.12	-0.4, 0.69


Data are presented as mean ± SD. The parameters were compared using IndependentSamples t test.

**Fig.1:**
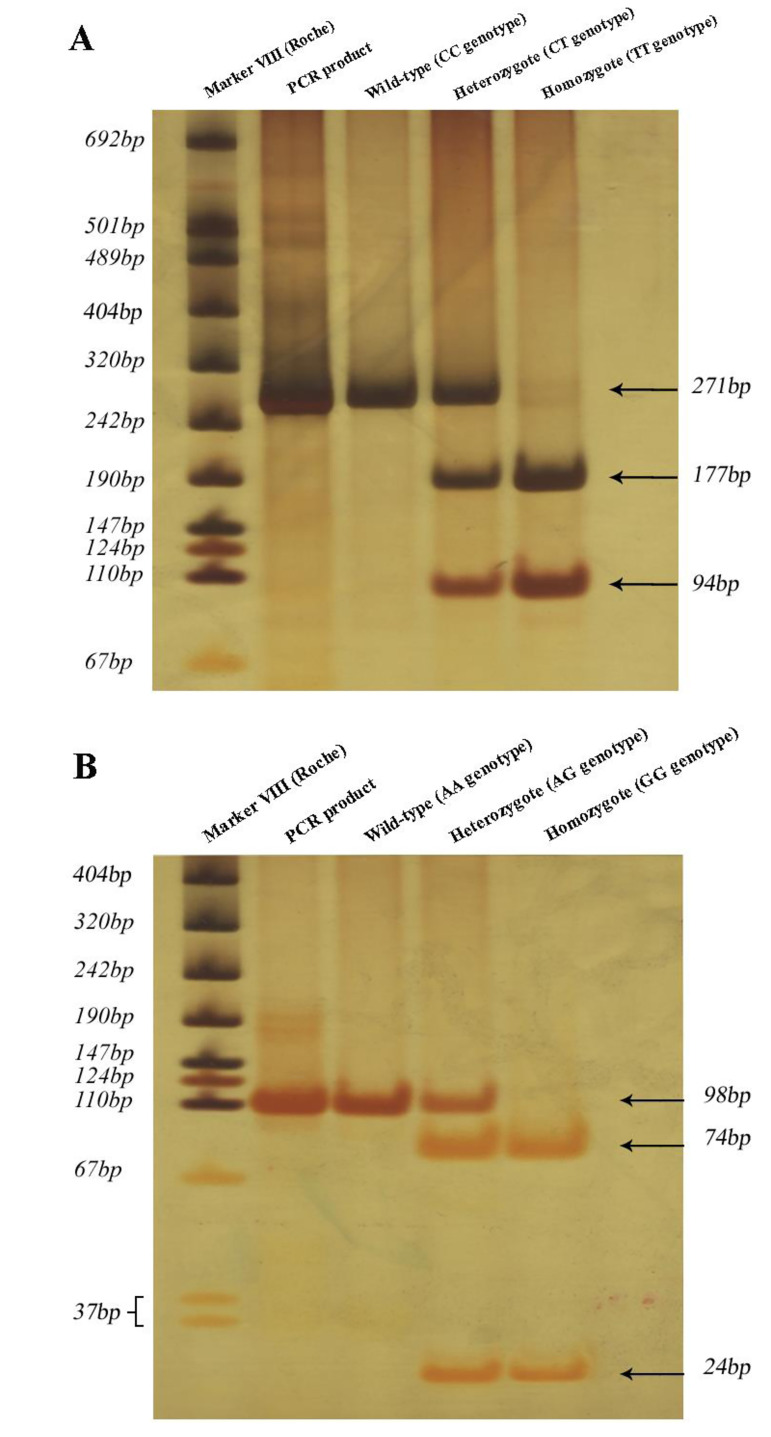
Polymorphisms of* MTHFR* and *MTRR* genes. A. Analysis of* MTHFR* C677T gene polymorphism with respect to digestion using HinfI restriction enzyme endonuclease to recognize 5'-GˆAGTC-3' sequence. Wild-type allelesof* MTHFR* gene were not digested with HinfI, but mutated alleles were digested using HinfI, resulting in two fragments. B. The result of PCR-RFLP for *MTRR* A66G gene polymorphism with respect to digestion using NdeI restriction enzyme endonuclease to recognize 5'-CAˆTATG-3' sequence. Wild-type alleles of *MTRR* gene were not digested with NdeI, but mutated alleles were digested using NdeI, resulted in two fragments. To detect fragments, we performed a 10% polyacrylamide gel with silver nitrate staining. PCR-RFLP; Polymerase chain reaction-restriction fragment length polymorphism.

Figure 1 shows the results of PCR-RFLP for MTHFR C677T and MTRR A66G gene polymorphisms with regard to digestion using HinfI and NdeI, respectively. 

Analysis of* MTHFR* gene polymorphism indicated that frequency of CC genotype in OAT men was higher than in men with normozoospermia (59.8 vs. 42.9%), while the frequency of CT genotype was lower in OAT men (46.8 vs. 34.3%). In addition, frequency of TT genotype was lower in OAT men (10.3 vs. 5.9%, Table 2).

As given in Table 2, analysis of *MTRR* gene polymorphism in normozoospermic and OAT men also revealed that frequency of AA genotype in normozoospermic group was higher than in OAT group (26 vs. 22%). Frequency of AG genotype in the two studied groups was almost identical (61 vs. 60.6%) and frequency of GG genotype in OAT group was higher than normozoospermic group (17.4 vs. 13%).

**Table 2:** Frequency of* MTHFR* and *MTRR* genotyping


Groups	MTHFR genotyping		
	CC	CT	TT

Normozoospermic group	33 (42.9)	36 (46.8)	8 (10.3)
Oligoasthenoteratozoospermic group	152 (59.8)	87 (34.3)	15 (5.9)
***MTRR* genotyping**			
	**AA**	**AG**	**GG**
Normozoospermic group	20 (26)	47 (61)	10 (13)
Oligoasthenoteratozoospermic group	56 (22)	154 (60.6)	44 (17.4)


Data are presented as n (%). Comparing between cases and controls was performed using Monte Carlo test.

According to Table 3, comparison of C (CC) and T carrier (CT+TT) in* MTHFR* C677T between normozoospermic and OAT groups showed that T carrier was conversely lower in OAT group than normozoospermic group (χ^2^- test=7.245, P=0.02). Additionally, our analysis did not show significant difference (χ^2^-test=1.079, P=0.53) in A (AA) and G carrier (AG+GG) from *MTRR* A66G between normozoospermic men and OAT men (Table 3). 

**Table 3:** Association between normozoospermic group and OAT group in* MTHFR* and *MTRR* SNPs


Groups	*MTHFR* SNP number		χ^2^-test, P value
	C carrier	T carrier	

Normozoospermic group	33	44	7.245, 0.02
Oligoasthenoteratozoospermic group	152	102	
***MTRR* SNP number **			
	**A carrier**	**G carrier**	
Normozoospermic group	20	57	1.079, 0.53
Oligoasthenoteratozoospermic group	56	198	


Comparing the cases with controls was performed using Monte Carlo test. OAT; oligoasthenoteratozoospermia and SNP; Single nucleotide polymorphism.

According to Table 4, the mean value of folate was 18.83 ± 17.1 ng/ml in normozoospermic group. So that 44.2% of the subjects had lower concentration than normal folate levels. In OAT group, the mean value of folate was 16.96 ± 14.2 ng/ml. Thus 36.1% of the subjects had lower concentration than normal folate levels. However, there was no significant difference in semen folic acid content between the two groups (P=0.47, OR=0.99, 95% CI=0.97-1.02). But, the concentration of vitamin B12 was slightly higher in normozospermic men compared to OAT men (522.6 ± 388.1 vs. 412.9 ± 303.6 pg/ml, P=0.058, OR=0.999, 95% CI=0.998-1.00). Low vitamin B_12_ concentrations were identified in 19.5 and 20.5% of the normozoospermic and OAT men, respectively

Furthermore, seminal folate and vitamin B12 concentrations were compared between C and T carrier in MTHFR C677T. They were also compared between A and G carrier in MTRR A66G, while none of them was statistically different (Table 5).

**Table 4:** Comparison of seminal folate and vitamin B_12_ concentration in oligoasthenoteratozoospermia and normozoospermic men


Groups	Folate concentration (ng/ml)	P value	Vitamin B12 concentration (pg/ml)	P value

Normozoospermic group	18.83 ± 17.1	0.47^*^	522.6 ± 388.1	0.058^*^^*^
Oligoasthenoteratozoospermic group	16.96 ± 14.2		412.9 ± 303.6	


Data are presented as mean ± SD. The parameters were compared using Independent-Samples t test. * ; OR: 0.99, 95% CI: 0.97-1.02 and **; OR: 0.999, 95% CI: 0.998-1.00.

**Table 5:** Comparison of seminal folate and vitamin B12 levels between C>T carrier in* MTHFR* C677T and A>G carrier in *MTRR* A66G


Concentration	*MTHFR* SNP number		P value
	C carrier	T carrier	

Folate (ng/ml)	16.88 ± 14.24	19.56 ± 17.59	0.31
Vitamin B12 (pg/ml)	427.51 ± 398.81	280.39 ± 191.14	0.17
** MTHFR SNP number**			
	**A carrier**	**G carrier**	
Folate (ng/ml)	16.71 ± 13.41	18.59 ± 16.65	0.54
Vitamin B12 (pg/ml)	506.89 ± 276.32	350.80 ± 360.95	0.25


Data are presented as mean ± SD. The parameters were compared using Fisher’s exact test.

## Discussion

Folate and other vitamins are vital for DNA synthesis and establishment of epigenetic modifications like DNA/ histone methylation (6). Spermatogenesis produces male haploid germ cells that involves distinct cellular and chromatin changes. Folate and normal activity of the corresponding enzymes play important role in nucleotide synthesis, methylation, maintenance of genomic integrity and prevention from DNA damage (12). Consequently, polymorphisms in folate metabolic genes have significant effect on spermatogenesis by inducing DNA hypomethylation and inducing mistakes in DNA repair, strand breakage and chromosomal abnormalities effect on the quality of sperm (16). There is substantial experimental evidence that folate metabolism pathway enzymes are essential for male spermatogenesis. Several SNPs which affect folate metabolism have been recognized, which in turn are associated with the cause of some defects (8). There was another study revealed that male mice lacking* MTHFR* suffered from severe reproductive defects. In these mice, spermatogenesis was failed during early postnatal development and resulted in total infertility (17).

But our analysis regarding the association of* MTHFR* C677T SNP and male infertility showed that T carrier is significantly lower in OAT men compared tonormozoospermic men. Liu et al. (3) found that the C677T mutation might affect the stability of RNA by performing a secondary structure of* MTHFR* mRNA sequence. Some studies showed that* MTHFR* C677T, A1298C and *MTRR* A66G polymorphisms are the risk factors with susceptibility to male infertility (4, 7, 18). However, the other study could not find any evidence for an association between reduced sperm counts and polymorphisms in enzymes involved in folate metabolism in the French population (19).

In the present study, no significant difference was observed in *MTRR* A66G variant between normozoospermic and OAT men. Similar studies were also developed by Kurzawski et al. (2) and Ni et al. (8), showing no significant difference in genotype frequencies of the gene polymorphisms in folate pathway between infertile and fertile men. They demonstrated that these genes in folate pathway were not risk factors for nonobstructive male infertility in the Polish and Chinese population (2, 8). In contrary, Liu et al. (3) proved using meta-analysis with trial sequential analysis that the genetic mutations in the folate-related enzyme genes played an important role in male infertility. However, the results of previous studies regarding this subject remain conflicting rather than definitive.

We found no statistical difference in seminal vitamin B12 and folate concentrations between normozoospermic and OAT men. But another study by Crha et al. (20) reported that folate and cobalamin were higher in seminal plasma from obstructive azoospermia than nonobstructive azoospermia patients.

The current study also demonstrated no association between* MTHFR* C677T and *MTRR* A66G polymorphisms with seminal vitamin B_12_ and folate concentrations innormozoospermic and OAT men. Similar study was also developed by Murphy et al. (21) while no significant correlation was observed between vitamin B_12_, folate, total homocysteine (tHcy) concentrations and any semen parameters in fertile and infertile men. They also found that infertile men had lower serum folate concentrations than fertile men, but there was no significant difference in red blood cell folate (RCF), B_12_ or tHcy. However, another study showed that adequate intake of vitamins B9 and B_12_ affects sperm parameters in men with different* MTHFR* polymorphisms, especially T allele genotypes (22).

As anticipated, OAT men had overall poorer semen parameters than normozoospermic controls. The results showed that infertile subjects had lower semen concentration, greater percentage of abnormal sperm morphology, higher percentages of non-motile sperm and more incidences of DNA fragmentation than fertile controls.

Some limitations of this study were low number of normozoospermic men in comparison with OAT men and absence of some demographic data regarding individuals that participated in the current study.

## Conclusion

The present study donot reveal the* MTHFR* C677T and MTRR A66G gene polymorphisms as risk factor for male factor subfertility in Iranian population. In addition, there is no statistical difference in seminal vitamin B_12_ and folate concentrations between normozoospermic and OAT men. Moreover, this study indicated no association between* MTHFR* C677T and *MTRR* A66G polymorphisms with seminal vitamin B_12 _and folate concentrations in normozoospermic and OAT men. However, larger sample size and well-designed studies are needed to compare the effect of other folate-related enzyme genes in the Iranian population. Larger sample size could generate better statistical results and sufficient data to confirm the improvement of secondary outcomes.
